# Can studies of pain help to bridge the gap between sensory and social impairments in autism?

**DOI:** 10.3389/fnhum.2013.00103

**Published:** 2013-03-28

**Authors:** B. M. Fitzgibbon, R. A. Segrave, P. B. Fitzgerald, P. G. Enticott

**Affiliations:** Faculty of Medicine, Nursing and Health Sciences, Monash Alfred Psychiatry Research Centre, The Alfred and Central Clinical School, Monash UniversityMelbourne, VIC, Australia

In May of 2013, the Diagnostic and Statistical Manual of Mental Disorders (DSM) will release its 5th edition. In this edition, the DSM-IV-TR categories of autistic disorder, Asperger disorder, childhood disintegrative disorder, and pervasive developmental disorder (not otherwise specified) will be combined into a single “autism spectrum disorder” (ASD) category. ASD will be diagnosed according to two symptom domains: “social communication impairment” and “restricted interests/repetitive behaviors.” The latter domain includes an additional feature that involves “hyper- or hypo-reactivity to sensory input or unusual interest in sensory aspects of environment” (Huerta et al., 2012). The relationship between the symptom domains that comprise ASD is not well-understood, and it has been suggested that these domains are largely independent (Dworzynski et al., 2009). Unusual sensory processing in ASD may, however, be associated with a disruption in higher-level social processes (Leekam et al., 2007), and therefore the social and sensory features of ASD may be inherently linked. In this general commentary, we propose that pain, as both a sensory and social experience, provides a potential paradigm with which to explore the relationship between sensory abnormalities and social impairments in ASD.

Physical pain is defined as the sensory and emotional experience associated with actual or potential tissue damage (IASP, 1994). Initial research suggests that individuals with ASD may have an insensitivity to physical pain (e.g., Nader et al., 2004). Although more research is needed to better characterize the experience of pain in individuals with ASD, such sensory abnormalities may be related to deficits in higher-order perceptual processes (Leekam et al., 2007). In particular, individuals with ASD may also demonstrate difficulty in the ability to vicariously experience the pain of others, a process that has been argued as integral to social perception (Keysers et al., 2010).

Indeed, several investigations have demonstrated that the processing of pain within a social context may be unusual in individuals with ASD. For instance, research suggests that when healthy controls observe another person experience physical pain (i.e., *empathy for physical pain*), similar (although not identical) neural networks are activated as if experiencing actual physical pain (Lamm et al., 2011). Among both control and ASD groups, however, this response is inversely correlated with alexithymia (a common feature of ASD; Bird et al., 2010). Furthermore, using transcranial magnetic stimulation, observing images of another person experience physical pain reveals no motor inhibition in individuals with ASD compared to controls (Minio-Paluello et al., 2009). In healthy subjects, an inhibited motor response is typically found (Avenanti et al., 2005).

Although not a sensory experience like physical pain, “social pain,” the experience of actual or potential damage to one's feeling of social connection or value, has also been shown to share overlapping neural networks with physical pain (Eisenberger, 2012). It has been hypothesized, that these shared neural mechanisms indicate that social pain is processed in the same way as physical pain. Moreover, it is suggested that this physiological overlap came about as an evolutionary adaptive process, whereby social pain mechanisms developed upon already existing processes for physical pain (MacDonald and Leary, 2005). In doing so, social pain is felt to encourage the development and maintenance of social bonds (Eisenberger, 2012). Thus, abnormalities within the physical pain network may impact upon the experience of social pain in ASD.

As expected, abnormalities in the neural experience of social pain have also been reported in individuals with ASD. For instance, two neuroimaging studies of social exclusion have found reduced activation within social pain brain regions identified in neurotypicals. Intriguingly, however, individuals with ASD reported levels of distress to the stimuli similar to that of controls (Bolling et al., 2011; Masten et al., 2011a). This suggests that people with ASD may not be impaired in their ability to recognize social pain, but that social pain may be processed differently. This is supported by a behavioral study of social pain in ASD; while there was no between group difference in the identification of social pain, people with ASD did not experience a subsequent reduction in mood that was found among controls (Sebastian et al., 2009).

Like empathy for physical pain, some brain regions active during the experience of social pain are also active when witnessing social pain in another (i.e., *empathy for social pain*) (e.g., Kross et al., 2011; Masten et al., 2011b). In a recent behavioral study exploring empathy for social pain in individuals with ASD, a similar empathy for social pain response to controls was observed when viewing *accidental* norm violations. In contrast, empathy for social pain was significantly reduced in ASD compared to controls when the norm violations were intentional (Paulus et al., 2013). This lack of behavioral resonance with another's social pain reflects the findings of reduced empathy for pain brain activity in individuals with ASD and its related features (Minio-Paluello et al., 2009; Bird et al., 2010), yet only in the condition requiring reflection on another person's mental state (i.e., identifying the intentionality of the behavior). Future imaging investigations should explore whether empathy for social pain recruits differential neural networks to controls, as is seen in social pain.

Taken together, these studies indicate that both physical and social pain are processed atypically in individuals with ASD. As these types of pain share overlapping neural substrates, sensory abnormalities in processing physical pain may, on some level, be associated with impaired social processing in ASD. Pain may therefore serve as a useful model in an attempt to bridge the gap between sensory abnormalities and impaired social function in ASD. This might contribute toward a more unified model of ASD. This is not only of potential importance in better understanding and managing ASD, but to psychiatric illnesses more generally where social impairments are becoming increasingly recognized (Cacioppo et al., 2007).
